# Supplementary material for: Genetic Structure and Colonization of North America by Depressaria depressana (Fabricius 1775) (Lepidoptera: Depressariidae) over 15 Years; Contrasts with Westward Expansion of Depressaria radiella (Goeze, 1783) over 160 Years
Source: Insects. 2022 Aug 31;13(9):789. doi: 10.3390/insects13090789 (PMC9504837; doi:10.3390/insects13090789)
Supplement: Supplementary file 1 [file insects-13-00789-s001.zip › insects-1866705-supplementary.pdf]

insects MDPI

Supplemental Table S1. All taxa, localities, BOLD IDs, and Genbank accession numbers used in this experiment.

Species	Continent	Country	BOLD ID	Genbank Accession
D. depressana	Europe	Austria	DEEUR156-11	KX042169
	Europe	Finland	LEFIC152-10	HM871998
	Europe	Finland	LEFIA1090-10	KF808477
	Europe	Switzerland	DEEUR049-11	KX042319
	Europe	Norway	LON2559-15	-
	Europe	Finland	LEFIB563-10	HM871445
	Europe	Finland	LEFIB562-10	HM871444
	Europe	Germany	ODOPE583-11	KX040162
	North America	Canada	RRINV3987-15	KT707656
	North America	Canada	MNAK285-10	HM887836
	North America	Canada	SMTPI8771-14	KT143113
	North America	Canada	GBGL4976-08	EU141359
	North America	Canada	GBGL26966-19	KT713819
	North America	Canada	AGAKH078-17	MG357416
	North America	Canada	SMTPS777-16	MG360114
	North America	Canada	SMTPD1365-13	KT139719
	North America	Canada	BARSH518-15	-
	North America	Canada	SMTPS599-16	MG360553
	North America	Canada	BARSG267-16	MG360136
	North America	Canada	BARSE545-16	MG361914
	North America	Canada	BARSG259-16	MG359965
	North America	Canada	ROUGE492-17	-
	North America	Canada	BARSD241-16	-
	North America	Canada	BARSC083-16	-
	North America	Canada	BARSI146-16	-
	North America	Canada	ELPCH679-17	-
	North America	Canada	MNAL583-10	HQ965238
	North America	Canada	MNAN645-13	KF808469
	North America	Canada	KSLEP022-14	KT138246
	North America	Canada	KSLEP021-14	KT146067
	North America	Canada	BARSM358-17	MG360133
	North America	Canada	BARSM752-17	MG361742
	North America	Canada	RRGCO536-15	MG362426
	North America	Canada	BARSD227-16	-
D. radiella	Europe	Austria	DEEUR158-11	KX042381
	Europe	Austria	LEATJ1038-15	-
	Europe	Austria	LEATC528-13	KM573322
	Europe	Finland	LEFIF849-10	HM875532
	Europe	Finland	LEFID175-10	HM872982

Europe	Germany	FBLMU176-09	HM391797
Europe	Germany	FBLMU177-09	HM391798
Europe	Germany	FBLMU703-09	HQ955266
Europe	Norway	LEPVM100-12	-
Europe	Norway	LEPVM101-12	-
Europe	Norway	LON2555-15	-
Europe	Russia	DEEUR477-15	MW201276
Europe	Switzerland	DEEUR045-11	KX042373
Europe	UK	CGUKA083-09	-
Europe	UK	CGUKC898-09	-
Europe	UK	CGUKD655-09	KX044169
Europe	Croatia	LON6919-18	-
Europe	Finland	LEFIF848-10	HM875531
Europe	Finland	LEFIB073-10	HM870982
Europe	Norway	GMNWK4200-14	-
Europe	Norway	GMNWL3227-14	-
Europe	Norway	GMNWK4187-14	-
North America	Canada	LALPA1354-12	KT127026
North America	Canada	LALPA1355-12	KT138815
North America	Canada	LBCW061-08	-
North America	Canada	MECB697-05	GU096427
North America	Canada	MEC086-04	GU095787
North America	Canada	RDLQD598-06	-
North America	Canada	RDLQD599-06	-
North America	USA	LNAUT2820-14	-
North America	USA	EHL851-12	-
North America	USA	RWWC908-12	-
North America	Canada	SMTPM6085-15	MG358908
North America	Canada	LOWCE394-06	KT138914
North America	Canada	LOWCE442-06	KT133300
North America	Canada	LOWCE437-06	KT129541
North America	Canada	LOWCE397-06	KT134163
North America	Canada	SMTPB5733-13	KT131398
North America	Canada	MEC171-04	GU095788
North America	Canada	MEC339-04	GU095789
North America	Canada	RDLQD600-06	-
North America	USA	LNAUT2821-14	-
North America	USA	RWWC1236-13	-
North America	USA	RWWC1336-14	-
North America	USA	RWWC1331-14	-
